# Uroperitoneum as an Atypical Source of Ascites: A Case Report

**DOI:** 10.7759/cureus.61498

**Published:** 2024-06-01

**Authors:** Leen Bakdash, Mary Ann Kirkconnell Hall, Patricia C Cheung

**Affiliations:** 1 Department of Medicine, Emory University School of Medicine, Atlanta, USA; 2 Division of Hospital Medicine, Emory University School of Medicine, Atlanta, USA

**Keywords:** urinary bladder, ascites, rare case report, uroperitoneum, spontaneous bladder rupture

## Abstract

Uroperitoneum secondary to spontaneous bladder rupture is a rare cause of ascites associated with significant morbidity and mortality. It can be difficult to detect and is often initially mistaken for other, more common etiologies.

We present the case of a 56-year-old female with a history of cervical cancer treated with chemotherapy and radiation, radiation proctitis, and diabetes mellitus who presented with subacute onset abdominal pain and distension, urinary retention, and nausea. She had been diagnosed with cervical squamous cell cancer 12 years prior to presentation and was successfully treated with two months of chemotherapy and radiation, and a presumed recurrence five years later was treated to remission with chemotherapy.

The golden-yellow appearance of her ascitic fluid during diagnostic paracentesis raised suspicion for urinary ascites that was confirmed by an elevated ascites-to-serum creatinine ratio and computed tomography (CT) cystography. Subsequent CT cystogram demonstrated leakage of contrast from the bladder with a 0.5 cm irregularity noted at the bladder dome, potentially representing the site of extravasation. A Foley catheter was placed at the time of admission with an immediate output of 1 L of fluid. Subsequently, her abdominal distension significantly improved, and her creatinine began to downtrend. Gynecologic oncology and urology were consulted and determined that she was not a candidate for surgical intervention given the significance of her bladder scarring. Positron emission tomography (PET)/CT was performed and revealed no active cancer. At the time of discharge, she had no episodes of emesis. Additionally, her creatinine had fallen to 1.0 mg/dl. She was discharged with a Foley catheter with plans to follow up with outpatient urology.

While relatively uncommon, uroperitoneum should be suspected in patients presenting with new-onset ascites who have risk factors for spontaneous bladder rupture such as pelvic irradiation. Uroperitoneum has a significant rate of mortality and morbidity. Ascites urea and creatinine studies, followed by a CT cystogram if these studies are abnormal, should be performed in any patient with risk factors for uroperitoneum. Patients should be managed with the placement of a Foley catheter and urology consultation for surgical evaluation.

## Introduction

Ascites occurs when fluid collects in the peritoneal cavity; while commonly associated with cirrhosis, it has a broad differential including portal hypertension and malignancy [1,2]. Uroperitoneum secondary to bladder rupture represents a rare but important addition to this differential. While bladder rupture is often characterized in the setting of trauma or iatrogenic injury, it can also occur spontaneously. The incidence of spontaneous bladder rupture is not well characterized but is believed to be between 1:126,000 and 1:50,000 [2].

Risk factors include chronic alcohol abuse, diabetes mellitus, pelvic malignancy, and pelvic irradiation [3]. Diagnosis is often delayed, with a median time to diagnosis of 48 hours. Spontaneous bladder rupture is, however, accompanied by a high risk of morbidity (~27%) and mortality (~18%), with 29% of these deaths occurring within 72 hours of presentation [2].

Given its association with malignancy, uroperitoneum is often mistaken for malignant ascites. Here, we describe a case of uroperitoneum initially mistaken for recurrent malignancy and ultimately identified by the appearance of the ascites.

## Case presentation

A 56-year-old woman with a past medical history of stage IIIB cervical squamous cell cancer, diabetes mellitus, and schizoaffective disorder presented to the emergency department with five days of non-bloody, non-bilious emesis and abdominal pain. Her symptoms began while traveling internationally. Upon the onset of her symptoms, she had also developed bloody diarrhea and fevers. She subsequently developed increasing abdominal distension, difficulty voiding, as well as exertional dyspnea and chest pain. 

The patient had been diagnosed with cervical squamous cell cancer 12 years prior to presentation and was successfully treated with two months of chemotherapy and radiation. Five years later, she had a presumed recurrence that was treated to remission with chemotherapy. She had most recently seen gynecologic oncology one month prior to presentation at which point her history and physical exam revealed no evidence of disease.

Most of the history was obtained from the patient's sister who served as her caretaker. The patient herself was minimally verbal at baseline. However, she had never been diagnosed with developmental delay, and her sister affirmed that the patient had always been developmentally comparable to her peers.

Clinical findings

On exam, the patient was afebrile and normotensive and in no acute distress. A cardiovascular exam revealed a previously undocumented 2/6 systolic murmur best heard at the upper right sternal border. She demonstrated bilateral lower extremity edema that was more pronounced in the right leg. Her abdomen was soft and non-tender but distended. Point-of-care ultrasound (POCUS) revealed ascites and bladder distension (Table 1). 

**Table 1:** Case timeline. CT: computed tomography; POCUS: point-of-care ultrasound; PET: positron emission tomography

Hospital day	Significant events
-12 years	The patient was diagnosed with stage IIIB cervical squamous cell cancer and treated with chemotherapy and radiation.
-7 years	The patient was diagnosed with presumed recurrence and treated with chemotherapy.
-5 days	The patient suddenly developed bloody diarrhea and non-bilious, non-bloody emesis.
0	She presented to the emergency department with nausea, vomiting, and abdominal pain. POCUS revealed ascites and bladder distension. CT scan of her abdomen/pelvis revealed bladder distention, omental stranding, and lymphadenopathy.
+1 day	Diagnostic paracentesis was performed. Symptoms improved with urinary catheterization.
+2 days	CT cystogram was performed.
+6 days	PET/CT was performed.
+7 days	Hospital discharge.

Diagnostic assessment

**Figure 1:**
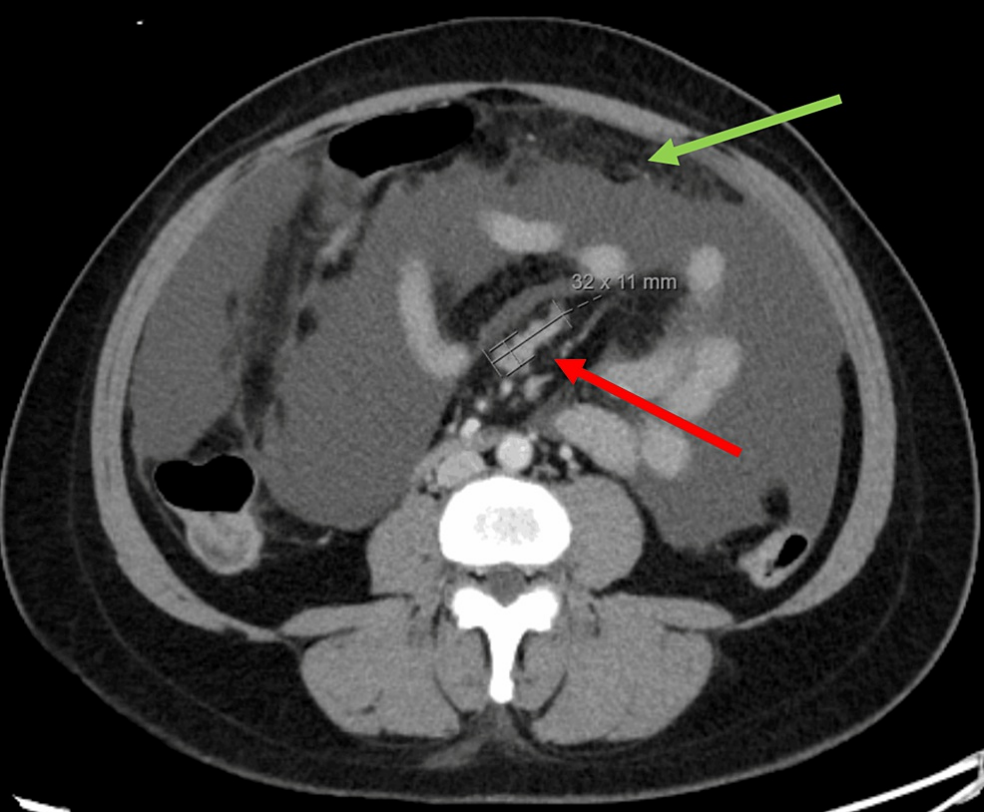
CT of the abdomen/pelvis demonstrating ascites, omental stranding (green arrow), and lymphadenopathy (red arrow). CT: computed tomography

At the time of admission, a computed tomography (CT) scan of her abdomen/pelvis (Figure 1) revealed bladder distension, omental stranding, and lymphadenopathy concerning for peritoneal carcinomatosis. Serum studies were notable for blood urea nitrogen of 40 mg/dl, creatinine of 5.3 mg/dl (baseline 1.1.-1.3 mg/dl), and lactate of 3 mmol/L. A diagnostic paracentesis was subsequently performed. Grossly, the ascitic fluid was golden yellow in color and translucent, raising suspicion for urinary rather than malignant ascites. The ascites creatinine was 20.8 mg/dl with a serum-to-ascites creatinine ratio of 3.92 mg/dl. The ascitic fluid white blood cell count was 10/mcl, and the serum ascites-albumin gradient was greater than 1.1 g/dl.

**Figure 2:**
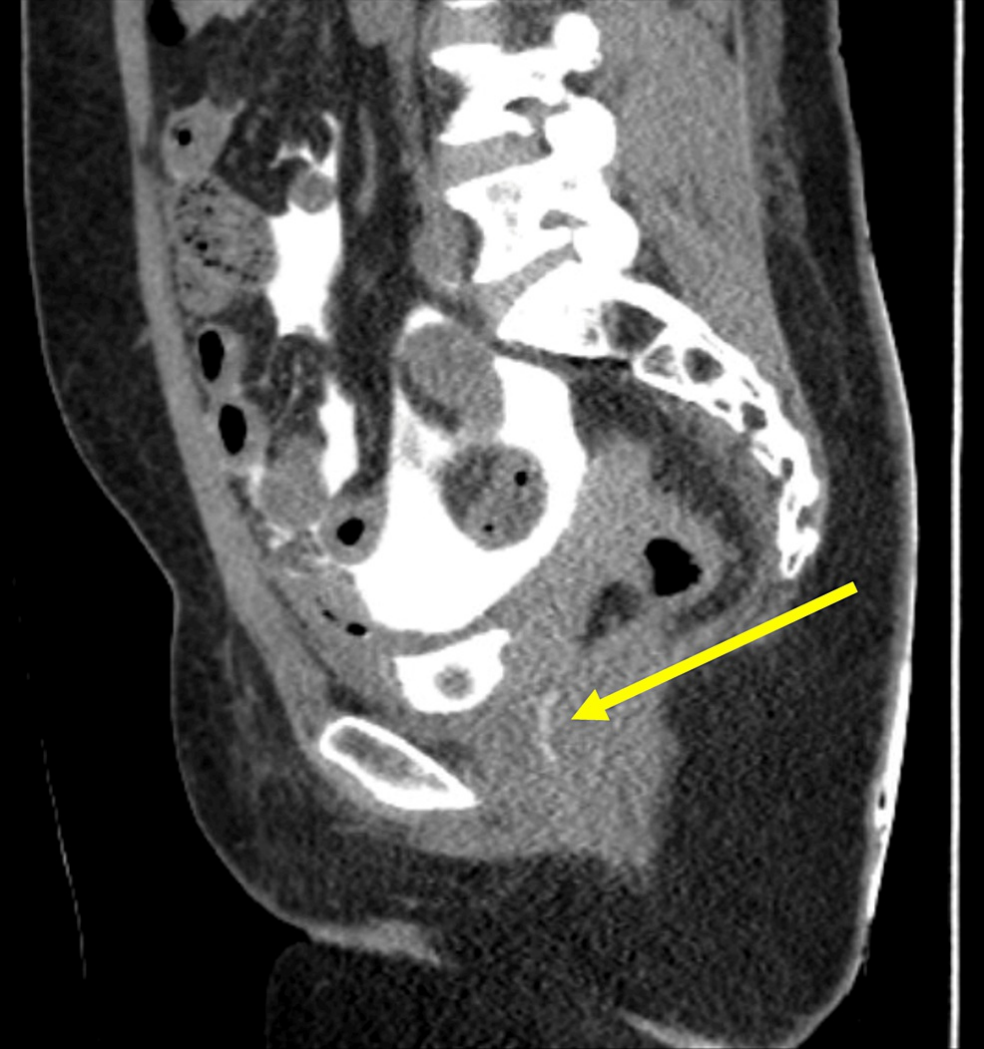
CT cystogram demonstrating leakage of contrast (yellow arrow) from the bladder into the vagina. CT: computed tomography

A subsequent CT cystogram (Figure 2) two days after she was admitted demonstrated leakage of contrast from the bladder with a 0.5 cm irregularity noted at the bladder dome, potentially representing the site of extravasation. The contrast was also seen in the vaginal canal.

Therapeutic intervention

A Foley catheter was placed at the time of admission with an immediate output of 1 L of fluid. Subsequently, her abdominal distension significantly improved, and her creatinine began to downtrend. Gynecologic oncology and urology were consulted and determined that given the significant bladder scarring she had sustained from pelvic irradiation, she was not a candidate for surgical intervention.

Follow-up and outcomes

Positron emission tomography (PET)/CT was performed and revealed no active cancer. Given the asymmetric lower extremity edema and recent travel, there was initial concern for deep vein thrombosis; however, an ultrasound of her legs was negative for thrombosis.

At the time of discharge, her chest pain had resolved, and she had no additional episodes of emesis. Her creatinine had fallen to 1.0 mg/dl. She was discharged with a Foley catheter and plans to follow up with outpatient urology for further management.

## Discussion

Uroperitoneum should be suspected in patients who present with ascites, urinary retention, and signs of acute kidney injury who have had a history of radiation, recent urologic or gynecologic procedure, or trauma. Many patients with uroperitoneum present with non-specific symptoms, the most common of which are unspecified abdominal pain and nausea/vomiting. Thus, clinicians should have a high index of suspicion for patients with these risk factors presenting with new ascites [2]. Though spontaneous bladder rupture is often diagnosed during surgery, ascites creatinine and urea studies as well as CT cystography can be used to diagnose this condition non-surgically [2,4]. Management of these patients may involve surgery or conservative management with bladder decompression, and urology should be consulted for their care [4,5].

In this case, the diagnosis of uroperitoneum was first suspected due to the golden-yellow appearance of the ascitic fluid, similar to urine. While there is minimal literature documenting the appearance of ascitic fluid in cases of uroperitoneum, there are known associations between the appearance of ascitic fluid and other diagnoses. For example, ascitic fluid with a dark yellow-green color is associated with bile leakage [6]. Turbid ascitic fluid is associated with spontaneous bacterial peritonitis and chylous ascites and has been shown to have a 46.9% sensitivity and 87.4% specificity for the diagnosis of spontaneous bacterial peritonitis [7,8]. In other reports of uroperitoneum, ascites has been reported to be transparent and yellow [9,10]. Further characterization of the appearance of ascitic fluid, specifically the shade of the fluid, in cases of uroperitoneum may be helpful in further evaluating the utility of fluid appearance in the diagnosis of uroperitoneum. 

The improvement in the patient's creatinine following catheterization is in line with pseudorenal failure in cases of uroperitoneum. Elevations in serum creatinine and blood urea nitrogen are often seen in patients with uroperitoneum; however, rather than reflecting impaired renal function, this is a consequence of the absorption of urine through the peritoneal membrane into the bloodstream [5]. In this patient with dyspnea, chest pain, recent prolonged immobilization during travel, and asymmetric lower extremity edema, one factor in the decision not to obtain a CT with contrast was a concern for renal impairment. Thus, distinguishing pseudorenal from true renal failure can be vital in guiding clinical decisions.

## Conclusions

Uroperitoneum is an important inclusion in the differential for ascites, especially in patients with a history of radiation to the cervix, uterus, or bladder. It has a significant rate of mortality and morbidity. Ascites urea and creatinine studies, followed by a CT cystogram if these studies are abnormal, should be performed in any patient with risk factors for uroperitoneum. Patients should be managed with the placement of a Foley catheter and urology consultation for surgical evaluation.
